# Bimanual examination for clot evacuation: a retrospective cohort study of women with postpartum haemorrhage after vaginal delivery

**DOI:** 10.1186/s12884-020-02916-w

**Published:** 2020-04-25

**Authors:** Pui Ru Koh, Daria Di Filippo, Andrew Bisits, Alec W. Welsh

**Affiliations:** 1 School of Women’s and Children’s Health, University of New South Wales, Randwick, NSW Australia; 2 Department of Obstetrics, Royal Hospital for Women, Sydney, NSW Australia; 3 Department of Maternal-Fetal Medicine, Royal Hospital for Women, Locked Bag 2000, Barker Street, Randwick, NSW 2031 Australia

**Keywords:** Postpartum haemorrhage, Uterine evacuation, Bimanual clot evacuation

## Abstract

**Background:**

Bimanual clot evacuation (BCE) is a simple clinical manoeuvre that may reduce need for surgical intervention in the management of severe postpartum haemorrhage (PPH). We sought to determine whether performing BCE in cases of severe PPH after vaginal birth reduces the need for surgical intervention.

**Methods:**

A retrospective chart review of women who delivered vaginally with a severe PPH between January 1, 2011 and December 31, 2014 in a single tertiary women’s hospital in Sydney, Australia was conducted. Severe PPH was classified as a blood loss ≥1000mls. The need for surgical management (including operating theatre uterine exploration or evacuation, intrauterine balloon tamponade, repair of significant trauma, uterine or internal iliac artery ligation, B-Lynch suture insertion or hysterectomy) was the primary outcome measure, as expressed by need for operating theatre utilisation.

**Results:**

From a cohort of 438, 149 women (34.0%) had BCE, of whom 29 (19.5%) required surgical management compared to 103 of 289 women with no BCE (35.6%); an odds ratio (OR) of 0.38 for BCE (confidence interval 0.20–0.72; *p* = 0.003). Early BCE (< 1 h of delivery) was associated with a further reduction in surgery (OR 0.24; confidence interval 0.08–0.70; *p* = 0.009) compared to late BCE (> 1 h of delivery). There was no reduction in estimated blood loss (*p* = 0.86) or blood transfusion (*p* = 0.71) with BCE.

**Conclusion:**

Our study suggests BCE reduces theatre utilisation in the context of severe PPH following vaginal delivery. Prospective trials are needed to determine whether BCE should be endorsed as a treatment modality for PPH post-vaginal delivery.

## Background

The World Health Organisation defines postpartum haemorrhage (PPH) as blood loss > 500 ml, or any amount resulting in haemodynamic instability after delivery [1]. Classified as severe if ≥1000mls, PPH remains the leading global cause of maternal mortality and morbidity [2], causing 140,000 deaths per year [3]. Our own institution in Australia has previously reported a severe PPH rate of 4.7% [4]. Women with severe PPH may require intensive care unit (ICU) admission, surgical interventions for bleeding control (e.g. uterine evacuation, intrauterine balloon tamponade, repair of cervical lacerations and other trauma, or more invasive therapy such as uterine or internal iliac artery ligation, B-Lynch suture insertion or hysterectomy), and transfusion of blood products [5]. Because the rate of PPH has been increasing in well-resourced countries, attention has been focussed on strategies to improve how PPH is managed on labour and delivery units [6–8].

Arrest of bleeding in the immediate postpartum phase is related to the ability of the uterus to fully contract with myometrial fibres acting as ‘physiological ligatures’ on the blood supply to the placental bed. In addition to mechanical haemostasis, local haemostatic (tissue factor, type-1 plasminogen activator inhibitor) and systemic coagulation (platelets, circulating clotting factors) factors are also activated to induce haemostasis [7, 8]. Any space-occupying tissue (clot or retained placenta or membrane) may prevent full uterine contraction, with surgical intervention needed to control the bleeding. Bimanual clot evacuation (BCE) may be a viable surgical intervention. BCE involves ‘cupping’ the fundus of the uterus with one hand whilst performing a vaginal examination to digitally break down and expel any blood clots [9]. This is hypothesised to enhance the effect of uterotonic agents on myometrial tissue by facilitating emptying of the uterine cavity. This procedure, generally performed under maternal analgesia obtained with nitrous oxide or a pre-existing epidural, is traditionally taught by more senior obstetricians and midwives, though has received limited academic attention. Of four contemporary national College PPH guidelines from the United Kingdom (RCOG), United States of America (ACOG), Australia and New Zealand (RANZCOG), and Canada (SOGC), none discuss the role of BCE [10], though a previous SOGC Clinical Practice Guideline did describe use of an initial exploration of the uterus, without a clear description of BCE [11]. The 2012 World Health Organisation (WHO) recommendations do mention the role of a similar procedure, bimanual uterine compression, as a temporizing measure for PPH until further definitive management can be applied, however this is a distinctly different procedure to BCE, where only physical pressure is applied for compression to aid haemostasis. Whilst this has shown potential benefit there are only a limited number of randomised controlled trials, with both a Cochrane Systematic Review and a more recent systematic review and meta-analysis being inconclusive [12, 13].

End-points for PPH that may be used to evaluate efficacy of management include volume of blood loss with associated physiological compromise and need for advanced surgical procedures that may require operative intervention. Given the lack of published information on this commonly performed and relatively simple procedure, our primary aim was to compare rates of surgical intervention among women receiving vs not receiving BCE in the setting of severe PPH post-vaginal delivery.

## Methods

A retrospective cohort study of management of severe PPH cases (≥1000mls) occurring between January 1, 2011 to December 31, 2014 at the Royal Hospital for Women in Sydney, Australia was undertaken. Ethics approval was obtained from the District Ethics Review Committee (13/076-LNR 13/POWH/418); all data were non-identifiable, and individual informed consent for participants was not required.

Participants were included in the study if they delivered vaginally, either spontaneous or assisted, and had severe PPH as defined by best obstetric estimate and quantitative blood loss where applied. Data was extracted from a state-wide obstetric clinical reporting system developed by the New South Wales (NSW) Department of Health Obstetrics Consortium (‘ObstetriX’; Meridian Health Informatics, Sydney, Australia). ‘ObstetriX ‘is used in 13 NSW Local Health Districts and in 61 maternity facilities across NSW. It is responsible for almost 60% of the electronic submissions to the NSW Perinatal Data Collection, and provides other functionality which assists in the documentation of obstetric episodes of care and in other reporting to Public Health Units, enabling hospitals and NSW Health to meet their statutory reporting requirements electronically. The database was filtered to generate the medical record numbers of only women who delivered vaginally and had PPH > 1 l during the study period. Maternal records of these women were then retrieved and manually reviewed for data collection. The lowest haemoglobin results were retrieved from the hospital computer pathology system using the generated medical record numbers (‘Powerchart’; Cerner Inc., North Kansas, USA).

Data collected included socio-demographic and obstetric characteristics along with delivery, neonatal and PPH management details. Socio-demographic characteristics collected were maternal age, body mass index, and model of care (public or private). Obstetric characteristics included parity, gestation (single or multiple) and previous PPH. Delivery details included labour onset (spontaneous or induced), mode of birth (spontaneous or assisted vaginal delivery), gestational age, epidural usage, duration of 3rd stage, and total estimated blood loss. Neonatal details included birth weight.

We undertook detailed physical review of clinical documentations regarding the management of PPH, which included time of birth, time of medical notification and of actual medical review, time of bimanual clot evacuation (if performed), type, timing of and total dose of uterotonic agents, additional operative intervention, need for transfusion of blood products, and lowest postpartum haemoglobin level. The latter was used as a surrogate marker of blood loss, given the known inaccuracy of visual blood loss estimation [14, 15].

The primary outcome for this study was the rate of significant surgical intervention; including need for uterine exploration or evacuation, the use of intrauterine balloon tamponade, repair of significant uterine, cervical or vaginal trauma, uterine or internal iliac artery ligation, B-Lynch suture insertion or hysterectomy. Given the additional costs, disruption to the mother:baby unit and potential associated complications we chose to use operating theatre utilisation as our marker for ‘significant’ surgical intervention. Secondary outcome measures were total blood loss, need for blood transfusion, lowest haemoglobin (Hb) level and use of uterotonic medications.

At this tertiary obstetrics referral hospital there were approximately 4200 births per year with a vaginal delivery rate of 70%, equating to 2940 vaginal births in a year. Given an estimated frequency of 5% for severe PPH, we anticipated approximately 150 women to undergo surgical intervention after PPH per year. Selecting surgical intervention as the primary outcome, and estimating that approximately one third of cases would have BCE, we calculated that a sample of 462 patients was needed for 90% power to detect a 50% difference from 25.0 to 12.5% at a 2-sided alpha level of 0.05, requiring study of cases over a 4-year period. Statistical analysis was performed using SPSS version 23 (IBM Corp, Armonk, New York, United States), and STATA version 16 (StataCorp, College Station, TX) for multivariable logistic regression. Continuous outcomes were assessed by t-test, or by multiple linear regression when there were potential confounding variables. Binary and categorical outcomes were assessed by Chi-squared test, or by logistic regression when there were potential confounding variables. The alpha level used for the tests is 0.05.

A subgroup analysis was performed for those who underwent ‘early’ BCE (within 1 h of delivery) versus later BCE, as we hypothesised that earlier emptying of the uterus would facilitate more effective use of other measures such as administration of uterotonic agents, thus reducing theatre utilisation.

### Study settings

The study was carried out in the Royal Hospital for Women (Sydney), a level-6 quaternary referral hospital, which is also a major teaching hospital affiliated to the University of New South Wales (UNSW).

## Results

There were 438 cases of severe PPH at the tertiary hospital between January 1, 2011 and December 31, 2014 (3.8% of vaginal births). Of these, 149 women (34.0%) underwent BCE. Socio-demographic, obstetric and birth characteristics are summarised in Table 1. Compared to those not receiving BCE, women receiving BCE were slightly younger and were less likely to experience a third stage length longer than 30 min.

**Table 1:** Socio-demographic, obstetric and delivery details

		Total cohort (*n* = 438)	BCE performed (*n* = 149)	BCE not performed (*n* = 289)	*p* value^1^
Maternal age (mean; years)		32.4	31.7	32.7	**0.03**
BMI > 30		29 (6.6%)	10 (6.7%)	19 (6.6%)	0.89
Public care (%)		95.4	95.3	95.5	0.92
Nulliparity		263 (60.0%)	82 (55.0%)	181 (62.6%)	0.12
Singleton pregnancy		427 (97.5%)	146 (98.0%)	281 (97.2%)	0.63
Previous PPH (%)		7.5	8.1	7.3	0.77
Birth weight (mean; grams)		3570.4	3591.0	3559.8	0.60
Spontaneous labour		285 (65.1%)	100 (67.1%)	185 (64.0%)	0.52
Spontaneous birth		280 (63.9%)	99 (66.4%)	181 (62.6%)	0.47
Delivery gestation:	Preterm (< 37 weeks)	26 (5.9%)	5 (3.4%)	21 (7.3%)	0.25
	Term (37–41 weeks)	325 (74.2%)	117 (78.5%)	208 (72.0%)	
	Post-term (> 41 weeks)	87 (19.8%)	27 (18.1%)	60 (20.8%)	
Prolonged 3rd stage (> 30 min)		66 (15.1%)	10 (6.7%)	56 (19.4%)	**< 0.001**

^1^Continuous variables were assessed by the t-test and categorical variables were analysed using the chi-squared test. *P* value of < 0.05 is considered statistically significant

Primary and secondary outcomes are summarised in Table 2. In our cohort of 438, 149 women (34%) underwent BCE, while the remaining 289 (66%) did not. In the BCE group, 19.5% needed surgery compared to 35.6% women without BCE (OR 0.38; 95% CI 0.20–0.72; *p* = 0.003). Of the 149 who had BCE, it was performed early in 104 women, and late in 45 women. Surgical intervention was required in 16 of the early BCE group (15.4%), and in 13 (28.9%) women who had it performed late (OR 0.24; 95% CI 0.08–0.70; *p* = 0.009).

**Table 2:** Primary and secondary outcomes

	Total Cohort (*n* = 438)	BCE Performed (*n* = 149)	BCE not performed (*n* = 289)	*p* value^1^
Surgical intervention	132 (30.1%)	29 (19.5%)	103 (35.6%)	**0.003**
*Uterine evacuation*	46 (10.5%)	24 (16.1%)	22 (7.6%)	**0.006**
*MROP*	64 (14.6%)	0 (0%)	64 (22.1%)	**< 0.001**
*Bakri Balloon*	11 (2.5%)	5 (3.4%)	6 (2.1%)	0.417
*Cervical repair*	1 (0.2%)	0 (0%)	1 (0.3%)	1.00
*Vaginal repair*	107 (24.4%)	21 (14.1%)	86 (29.8%)	**< 0.001**
*Vaginal packing*	2 (0.5%)	1 (0.7%)	1 (0.3%)	1.00
*Vaginal haematoma drainage*	2 (0.5%)	0 (0%)	2 (0.7%)	0.55
*Uterine artery ligation*	1 (0.2%)	0 (0%)	1 (0.3%)	1.00
*Uterine artery embolization*	1 (0.2%)	1 (0.7%)	0 (0%)	0.34
*B Lynch suture insertion*	1 (0.2%)	0 (0%)	1 (0.3%)	1.00
*Hysterectomy*	1 (0.2%)	1 (0.7%)	0 (0%)	0.34
Surgical intervention with early BCE (*n* = 104) versus late BCE (*n* = 45)	–	16 (15.4%) 13 (28.9%)	–	**0.009**
Blood loss (mean in mL)	1626.0	1633.3	1622.3	0.86
Blood transfusion (packed cell unit)	2.5	2.6	2.5	0.71
Lowest postpartum haemoglobin (g/L)	94.4	93.3	95.0	0.36

^1^Continuous variables were assessed by the t-test and categorical variables were analysed using the chi-squared test. *P* value of < 0.05 is considered statistically significant

Multivariate regression analysis was performed using surgical intervention as the dependent variable, and BCE, the use of uterotonic agents, total blood loss and length of the third stage and primiparity as independent variables*. When analysed after adjusting for confounders, the difference between the 2 groups were still statistically significant. The goodness of fit test for this model using the Hosmer-Lemeshow test (*p* = 0.28) showed adequate fit.

BCE was not associated with a reduction in total blood loss (*p* = 0.86), blood transfusion (*p* = 0.71) or lowest postpartum haemoglobin (*p* = 0.36). The average time taken for medical officer notification was longer in the BCE group than in the non-BCE group (39.83 vs 17.78 min, *p* = 0.003). The average times taken for actual medical review in the BCE and non-BCE groups following notification were 4.2 and 3.0 min, respectively. There was no association between speed of attendance and the performance of BCE (*p* = 0.47).

$$ \ast \pi (surgicalmx)/1-\pi (surgicalmx)= e\alpha +\beta 1 BCE+\beta 2 prostaglandins+\beta 3 totalbloodloss+\beta 4 prolonged3 rdstage+\beta 5 ergometrine+\beta 6 primipar $$

A variety of surgical procedures were documented in the notes, as seen in Table 2. The commonest procedure was repair of genital tract trauma, occurring in 107 out of the 438 cases (24.4%). Manual removal of placenta (MROP) was performed in 14.6% and uterine evacuation in 10.5%. Other procedures used were: Bakri balloon 2.5%; vaginal packing 0.5%; vaginal haematoma drainage 0.5%; bilateral uterine artery ligation, bilateral uterine artery embolization, hysterectomy and B-Lynch suture, each one case (0.2%). Uterine evacuation, MROP and repair of genital tract trauma were utilised more in the non-BCE group, reaching statistical significance (*p* < 0.001).

Table 3 shows uterotonic drug usage following routine intramuscular Oxytocin and association with bimanual clot evacuation. Use of Ergometrine was statistically higher in the BCE than non-BCE groups (85.2 vs 56.4%; *p* < 0.001). There were also significantly higher proportion of patients requiring additional ergometrine doses, misoprostol and PgF2α in the BCE group. There was no statistically significant difference in the use of oxytocin infusion between the groups (100 vs 97.9%, *p* = 0.10). A sub-group analysis demonstrated that Oxytocin infusion administered after BCE was associated with a statistically significant, yet modest, reduction in blood loss (1419.5 vs 1676.7 ml, *p* = 0.001). Misoprostol given after BCE resulted in a statistically significantly higher haemoglobin level (92.3 vs 80.0 g/L; *p* = 0.001). Misoprostol had a statistically significant influence on the lowest postpartum haemoglobin for those who received BCE (90.5 vs 96.2 g/L; *p* = 0.015) whereas it did not have the same effect in those who did not receive BCE (94.5 vs 98.0 g/L; *p* = 0.482).

**Table 3:** Uterotonic drug usage and usage of BCE

	Total cohort (*n* = 438)	BCE performed (*n* = 149)	BCE not performed (*n* = 289)	*p* value^1^
Oxytocin infusion	432 (98.6%)	149 (100%)	283 (97.9%)	0.10
Ergometrine	290 (66.2%)	127 (85.2%)	163 (56.4%)	**< 0.001**
Ergometrine-additional dose	135 (30.8%)	76 (51.0%)	59 (20.4%)	**< 0.001**
Misoprostol	186 (42.5%)	93 (62.4%)	93 (32.2%)	**< 0.001**
PgF2α	10 (2.3%)	8 (5.4%)	2 (0.7%)	**0.004**

^1^Continuous variables were assessed by the t-test and categorical variables were analysed using the chi-squared test. *P* value of < 0.05 is considered statistically significant

## Discussion

Our findings suggest that, among women who experience severe PPH after vaginal delivery, BCE may be associated with a lower risk of operating theatre utilisation (OR 0.38; 95%CI 0.20–0.72; *p* = 0.003). Despite no associated reduction in total blood loss, need for blood transfusion, lowest Hb level or use of uterotonic medications (the secondary outcomes), these are highly relevant findings given the potential for adverse surgical consequences. However, these adverse consequences including direct procedural complications, disruption of the newly formed family unit and financial factors were not directly addressed in this work. Surgical interventions for PPH include uterine cavity tamponade, compression sutures, uterine or internal iliac artery ligation, and hysterectomy; all recommended worldwide [16–20], each with potential complications.

Ergometrine, Misoprostol and PgF2α were used more in the BCE group. This may reflect the severity of uterine atony, necessitating the use of BCE and all uterotonic agents, or that, having emptied the uterus, the treating clinician was more confident to continue treating with uterotonics rather than resorting to surgical intervention. Reduced blood loss when oxytocin infusion was given after BCE, and higher postpartum haemoglobin level when misoprostol was administered after BCE, illustrate how emptying the uterus allows medications to work more effectively on the myometrium. The use of the specific uterotonics was also in accordance with the current PPH local operating procedures within our institution throughout the study period. This did not include the use of tranexamic acid; which was not used at this time. However, at time of writing of this manuscript, we have released a new guideline in accordance with the updated NSW Health Policy Directive which involved early use of tranexamic acid (TXA), prior to PgF2α. Average times for medical officer attendance was after delivery was significantly higher in the BCE group. We acknowledge the potential that later review may preference cases more refractory to strategies other than BCE, which was generally initiated following medical officer attendance.

Of 132 patients with surgical haemostasis, 35% had uterine evacuation, 49% needed MROP and 81% required repair of genital tract trauma, illustrating the co-existence of multiple aetiological factors. The non-BCE group required statistically significantly more MROP. In the setting of retained placenta, clinical practice would be to proceed directly to the operating theatre for manual removal of the placenta, rather than undergoing additional examination in the delivery suite room for BCE. The reason for this is that BCE in the setting of retained placenta could not be a definitive therapy. The finding of more tear repairs in the non-BCE group likely reflects that in those cases, genital tract trauma was thought to be the main contributor of the bleeding, hence BCE was not performed.

Although we could find no published literature relating BCE with subsequent need for surgery, a French population-based study looking at components of PPH management showed that delaying bimanual uterine examination by more than 20 min after delivery increased the risk of severe PPH by 1.8 times, compared to performing it within 10 min of delivery [21]. The published literature on severe PPH mostly comprises retrospective audits assessing outcomes and complications of surgical interventions with sample sizes less than 200 [20–24]. The current study is a substantial single tertiary centre retrospective study on management and outcome of severe PPH, providing detailed information on maternal, pregnancy, labour and postpartum characteristics. Epidemiological studies have found a temporal increase in PPH analysing data from large perinatal databases, lacking detailed information on the management of labour [25].

There are several limitations to our current study. A theoretical disadvantage of this single center study is that, despite the use of a single robust state-wide database for all obstetrical outcomes, it relies on precise data input by staff; as is the case for the bulk of such retrospective studies. However, this effect is unlikely to be significant given the extensive cross-referencing between the medical files and the obstetric database during data collection. As with all minor procedures, we cannot exclude the possibility that BCE were performed but not documented, though, given the invasive nature of this procedure and the contemporary culture for informed consent and documentation, we consider this unlikely. Lowest postpartum haemoglobin was used as a marker for blood loss, given the unreliable nature of visual blood loss estimation. We acknowledge the potential for overestimation of blood loss occurring with excessive intravenous fluid administration. Collector bags used in certain European countries to accurately quantify the amount of blood loss [26], are not available in our institution. We acknowledge also that with this retrospective evaluation we were not able to determine at what stage of blood loss BCE was performed, and whether the comparator group received non-BCE intervention at an equivalent stage of bleeding. This is a deficiency that could be addressed in further prospective evaluation. Our paper includes several secondary outcomes and we acknowledge the potential for type 1 error. Therefore these need to be interpreted with caution, warranting further prospective evaluation. As this study was retrospective, we were not able to gain information regarding the contemporaneous skill and experience at performing BCE of the medical operators managing these cases of PPH. This should also be the subject of future evaluation.

Endometritis and patient discomfort due to bimanual examination were not formally assessed. In our institution, prophylactic intravenous antibiotics were routinely administered after BCE to prevent infection. Future studies could assess the complications of bimanual examination and the associated patient perspectives, particularly in those without epidural anaesthesia. Other limitations of the study are intrinsic to its retrospective design and single centre nature, potentially limiting external applicability, requiring further prospective evaluation.

## Conclusion

We believe this study to be the first evaluating the impact of BCE on management of severe PPH, showing an associated reduced need for surgical intervention. The data generated could form the basis for a prospective randomised controlled trial to more clearly delineate the role of BCE in management of severe PPH. Should this effect be consistent, we would recommend incorporation of BCE into PPH management algorithms; being a simple first line intervention with the potential to reduce the need for surgical intervention. Further prospective studies are indicated to clarify its role in clinical management and any potential complications.

## Supplementary information

**Additional file 1.** Uterotonic drugs- first and additional doses. *1st Oxytocin dose = 10 units IM; Additional dose = Oxytocin 10 units/hour intravenous infusion. 4/5 who did not receive Oxytocin IM was given Oxytocin infusion.. **PGF2α dose = 3 mg

## Data Availability

The dataset used and analysed during this study is available from the authors on reasonable request.

## Abbreviations

ACOG: The American Congress of Obstetricians and Gynecologists
BCE: Bimanual clot evacuation
Hb: Haemoglobin
MROP: Manual removal of placenta
PPH: Postpartum haemorrhage
RCOG: Royal College of Obstetricians and Gynaecologists
RANZCOG: Royal Australian and New Zealand College of Obstetricians and Gynaecologists
SOGC: The Society of Obstetricians and Gynaecologists of Canada
