# Supplementary material for: Bimanual examination for clot evacuation: a retrospective cohort study of women with postpartum haemorrhage after vaginal delivery
Source: BMC Pregnancy Childbirth. 2020 Apr 25;20:245. doi: 10.1186/s12884-020-02916-w (PMC7183670; doi:10.1186/s12884-020-02916-w)
Supplement: Supplementary file 1 — Additional file 1. Uterotonic drugs- first and additional doses. *1st Oxytocin dose = 10 units IM; Additional dose = Oxytocin 10 units/hour intravenous infusion. 4/5 who did not receive Oxytocin IM was given Oxytocin infusion.. **PGF2α dose = 3 mg [file 12884_2020_2916_MOESM1_ESM.docx]

Additional file 1: Uterotonic drugs- first and additional doses

| Drug | First dose given | Additional dose given |
| --- | --- | --- |
| Oxytocin* | 433/438 98.9 % | 428/433 98.8 % |
| Ergometrine  125 mcg IV  125 mcg IM  250 mcg IV  250 mcg IM  500 mcg IV  500 mcg IM | 290/438 66.2 %  4/290 1.4 %  0/290 0.0 %  46/290 15.9 %  235/290 81.0 %  1/290 0.3 %  4/290 1.4 % | 135/290 46.6%  5/135 3.7 %  1/135 0.7 %  114/135 84.4 %  13/135 9.6 %  2/135 1.5 %  0/135 0.0 % |
| Misoprostol  400 mg  800 mg | 186/438 42.5%  3/186 1.6 %  183/186 98.4 % | none |
| PgF2α** | 10/438 2.3 % | none |

*1^st^ Oxytocin dose= 10 units IM; Additional dose= Oxytocin 10 units/hour intravenous infusion. 4/5 who did not receive Oxytocin IM was given Oxytocin infusion.

**PGF2α dose= 3mg
